# Respiratory Tract Microecology and Bronchopulmonary Dysplasia in Preterm Infants

**DOI:** 10.3389/fped.2021.762545

**Published:** 2021-12-13

**Authors:** Tong Sun, Haiyang Yu, Jianhua Fu

**Affiliations:** ^1^Department of Pediatrics, Shengjing Hospital of China Medical University, Shenyang, China; ^2^Department of Neurology, Shengjing Hospital of China Medical University, Shenyang, China

**Keywords:** microecology, bronchopulmonary dysplasia, preterm infants, lung development, gut-lung axis, microbiota

## Abstract

Bronchopulmonary dysplasia (BPD) is a severe respiratory complication in preterm infants. Although the etiology and pathogenesis of BPD are complex and remain to be clarified, recent studies have reported a certain correlation between the microecological environment of the respiratory tract and BPD. Changes in respiratory tract microecology, such as abnormal microbial diversity and altered evolutional patterns, are observed prior to the development of BPD in premature infants. Therefore, research on the colonization and evolution of neonatal respiratory tract microecology and its relationship with BPD is expected to provide new ideas for its prevention and treatment. In this paper, we review microecological changes in the respiratory tract and the mechanisms by which they can lead to BPD in preterm infants.

## Introduction

The etiologies of bronchopulmonary dysplasia (BPD) are numerous, including prenatal and postnatal risk factors. Prenatal risk factors mainly include genetic susceptibility, intrauterine growth retardation, perinatal asphyxia, and chorioamnionitis. Postnatal risk factors mainly include mechanical damage, hyperoxia/oxidative stress, inflammation, infection and patent ductus arteriosus (1). However, the changes in the microecology of the respiratory tract may also lead to BPD (2, 3). In recent years, the microecological composition of the respiratory tract and its interaction with respiratory diseases has become a hotspot of research. Various factors are known to affect the colonization and evolution of respiratory tract microecology during the neonatal period, including delivery mode and feeding patterns (4, 5). And the changes of intestinal microecology could also affect the respiratory tract microecology by lung-gut axis and then cause BPD (6, 7). The microecological balance helps to maintain the stability of the microenvironment in the body, but its composition and content do not remain constant. Disturbance of this balance by adverse factors either *in vivo* or *in vitro* may lead to disease. In this paper, we review microecological changes in the respiratory tract and the mechanisms by which they can lead to BPD in preterm infants. The main results of included studies can be seen in Table 1.

**Table 1:** Characteristics and main results of included studies about the changes of respiratory tract microecology.

**References**	**Study design**	**Main results**
Payne et al. (8)	Cohort study [*n* = 55; PMA: mean, 26.2 week (range, 23–30 week)]	*Ureaplasma* was a risk factor of BPD or death. (OR = 4.80, 95% CI: 1.15–20.13)
Mourani et al. (9)	Cohort study (*n* = 10; PMA: 24–27 week)	*Staphylococcus* spp., *U. parvum*, and *U. urealyticum* were the most frequently identified dominant organisms, but *Pseudomonas* spp., *Enterococcus* spp., and *Escherichia* spp. were also present.
Lohmann et al. (10)	Cohort study (*n* = 25;PMA: 24–32 week)	Neonates with BPD had lower bacterial diversity, with increased *Firmicutes* and decreased *Proteobacteria*.
Lal et al. (11)	Cohort study (*n* = 51;Mean PMA: ELBW infants, 24.5 ± 0.2 week; full-term infants, 38.3 ± 2.2 week; infants with established BPD, 37.6 ± 1.5 week)	Infants with BPD was characterized by increased *Proteobacteria* and decreased *Firmicutes* and *Fusobacteria*. *Lactobacillus* was less abundant in infants with BPD.
Imamura et al. (12)	Case-control study (*n* = 169;PMA: 23–29 week)	*Corynebacterium species* were more frequently detected in the severe BPD than nonsevere BPD infants (*p* = 0.03).
Wagner et al. (13)	Cohort study (*n* = 152; Mean PMA: mild BPD, 25.69 ± 1.3 week; moderate BPD, 25.61 ± 1.63 week; severe BPD, 25.4 ± 1.56 week)	Preterm infants with severe BPD exhibited greater bacterial community turnover with age, acquired less *Staphylococcus* after birth, and had a higher initial relative abundance of *Ureaplasma*.

## Respiratory Tract Microecology

### Origination and Composition of Respiratory Tract Microecology in Neonates

The microecological environment of both the upper and lower respiratory tracts consists of numerous types of microbes such as bacteria, fungi, and viruses. The colonized microorganisms can be detected in the neonatal respiratory tract after birth. The main sources of these microorganisms include the amniotic fluid inhaled by the fetus, microbiome transferred in the placenta, the influence of vaginal microecology during vaginal delivery, the inhalation of air after birth, and the downward dissemination of nasopharyngeal and oral secretions (14). However, there is still controversy about the microbial status in amniotic fluid and placenta. The studies of Aagaard et al. showed that most placentas contained detectable bacterial DNA and they found communities dominated by *Escherichia coli* and a few other groups (15, 16). Collado et al. (17) also cultured bacteria successfully from placental tissues taken from women who had healthy pregnancies. Researchers have also found bacteria in amniotic fluid (18). But it is extremely difficult to do studies of low biomass samples such as placenta and amniotic fluid, which are susceptible to contamination during researches. Lauder et al. thought that comparison of placenta samples with contamination controls didn't provide evidence for a distinct placenta microbiota. In their study, they compared bacterial DNA recovered from placental samples from six uncomplicated deliveries at term to contamination controls, both the placental samples and contamination controls yielded quite low values, with no clear signal in the placental samples above negative controls (19).

The common dominant bacteria in the upper respiratory tract include *Moraxella, Dolosigranulum, Corynebacterium, Streptococcus*, and *Staphylococcus* in the *Firmicutes* phylum (20). Although early researches believed that the lower respiratory tract was sterile, recent studies have demonstrated otherwise. While most microorganisms in the lower respiratory tract originate in the upper respiratory tract, the latter lacks *Corynebacterium* and *Dolosigranulum*, among others (21). Generally, the composition of the lung microbiota at the phylum level is predominated by *Firmicutes* and *Proteobacteria* after birth (13). In addition, the lungs contain some microbiota that are absent in the upper respiratory tract, such as the gram-negative bacterium *Tropheryma Whipplei* (22).

### Influence Factors of Respiratory Tract Microecology in Neonates

The microbiome of the neonatal respiratory tract is affected by many factors. Different mode of delivery will affect the microbiome of neonates at birth. Vaginal delivery neonates are more likely to be affected by vaginal flora, the microbiome of neonates is mainly dominated by *Corynebacterium* and *Dolosigranulum*, not *Staphylococcus*, and the colonization of *Lactobacillus* increases. Cesarean delivery neonates are mainly affected by skin flora, and more likely to reduce the colonization level of protective flora, including *Corynebacterium* and *Dolosigranulum*. But the colonization of *Propionibacterium* and *Streptococcus* are increased. The difference caused by different mode of delivery will disappear at six weeks after birth and will be replaced by a more typical microecology of the body (4, 23).

Biesbroek et al. reported that different feeding modes can also affect the composition of neonatal respiratory tract microecology. At 6 weeks of age, there was a significant difference in the composition of overall microflora between breast feeding and formula feeding infants. Breastfeeding infants had more *Lactobacillus, Dolosigranulum*, and *Corynebacterium* in their respiratory tracts, but had fewer *Staphylococcus* and anaerobic bacteria, such as *Prevotella* and *Veillonella*. Formula feeding infants had higher levels of anaerobic bacteria, *Rothia, Gemella*, and *Granulicatella* in their respiratory tracts (5). Biesbroek et al. also found that the length of breastfeeding was positively correlated with the colonization levels of *Corynebacterium* and *Dolosigranulum*. Breastfeeding can improve the colonization of respiratory tract flora and contribute to the increase of beneficial bacteria to reduce asthma and neonatal respiratory tract infection. However, with the addition of complementary food, the difference in respiratory tract flora caused by different feeding modes disappeared gradually at 6 months of age (24–27).

Amniotic fluid is not sterile, maternal microbiome will also affect the respiratory tract microecology in neonate. Romero et al. (28) reported that the microorganisms cultured from amniotic fluid could also be detected in the vagina were UU, *E. coli*, and *Streptococcus agalactiae*, suggesting intra-amniotic infections are often the result of an ascending invasion. However, some scholars think the “*in utero* colonization hypothesis” is not tenable. They think most studies used molecular approaches with an insufficient detection limit to study “low-biomass” microbial populations, lacked appropriate controls for contamination, and failed to provide evidence of bacterial viability (29).

The use of broad-spectrum antibiotics can greatly affect the composition of intestinal microbiome, reduce its biodiversity, and delay colonization for a long period after administration. The use of antibiotics can also affect respiratory microflora directly or indirectly, but the long-term effects in infancy on the maturation of respiratory microflora remains uncertain (30). Host genes can also regulate the composition of human microbes. The microbiome itself can be thought of as a complex phenotype whose composition is influenced by environmental and genetic factors. Igartua et al. (31) reported that respiratory tract microbiome was affected by host genotypes at many gene loci, suggesting that the host innate immunity and the expression of genes in mucosal immunity pathways played an important role in respiratory tract microbiome formation. In addition, air pollution, vaccination, and seasons can also influence the composition of respiratory tract microecology (32–34).

### Diversity of Respiratory Tract Microecology

The microecological diversity of the respiratory tract refers to the number of microbial species colonized in the respiratory tract, which is usually expressed in terms of the Shannon diversity index. This index represents the number of species detected in the sample and their relative abundance distribution. A high Shannon diversity index usually represents a community with rich species and relatively uniform distribution, while a low Shannon diversity index usually represents a community that has few species and may be dominated by a few specific bacteria. The respiratory tract of a healthy newborn is colonized by a variety of microorganisms, such as *Moraxella, Dolosigranulum, Corynebacterium, Streptococcus*, and *Staphylococcus*. Studies have shown that the main colonized bacteria in the newborn respiratory tract are *Staphylococcus* and *Streptococcus*. In the days following birth, the species of microorganisms in the respiratory tract are constantly enriched, and communities such as *Moraxella* and *Haemophilus* of the phylum *Proteobacteria* begin to appear, gradually becoming the dominant bacteria (35). This rich community of species serves to maintain the stability of the internal environment, but disease status can affect the number of microbial species. Indeed, the microecological diversity of the respiratory tract decreases in premature infants with BPD (36).

### Evolution of Respiratory Tract Microecology

The microecological composition of the respiratory tract and the relative abundance of species within the respiratory tract also change during the process of growth and development in newborns—a process termed microecological evolution. Under normal circumstances, the evolution of respiratory tract microecology during the neonatal period is mainly characterized by a transition from *Staphylococcus*-dominated community to a *Corynebacterium*- or *Dolosigranulum*-dominated community, and finally to a *Moraxella*-dominated community (35). Similarly, disease may affect the evolution of respiratory tract microecology. Evolution of the microbial community is known to be accelerated in preterm infants with BPD (13).

## Respiratory Tract Microecology and BPD in Preterm Infants

### Diversity of Respiratory Tract Microecology and BPD

Lohmann et al. reported that within 24 h after endotracheal intubation, the number of respiratory microflora species and Shannon diversity index decrease in newborns with BPD, when contrasted with those without BPD. The authors of this study speculated that decreased diversity of the microbial community, rather than differences in inflammatory mediators, may be a significant factor in the occurrence of BPD. As reported previously, their study demonstrated that microbiome diversity is associated with host health. They included 25 preterm infants who underwent endotracheal intubation within 24 h after birth, 10 of whom developed BPD. The authors also observed that, when compared with the relatively stable and diverse flora in the non-BPD group, the relative abundance of *Firmicutes* exhibited an upward trend over time in infants with BPD, while that of *Proteobacteria* exhibited a downward trend. *Acinetobacter* was the dominant bacterium in both the BPD and non-BPD groups, although the relative abundance of *Acinetobacter* exhibited a downward trend in the BPD group over time, while that of *Staphylococcus* and *Klebsiella* exhibited an upward trend over time. The study suggested that the presence of *Acinetobacter* influences the subsequent evolution and colonization of the respiratory flora (10). In contrast to the findings of Mourani et al. (9), most of the samples obtained within 72 h after birth indicated inadequate bacterial DNA quantity. Lohmann et al. detected multiple bacterial floras in the early respiratory tract secretions, indicating that microbial colonization had occurred in the respiratory tract after birth.

The results of a prospective cohort study conducted by Lal et al. (11), which included 23 extremely low birth weight infants, 10 full-term infants, and 18 newborns with confirmed BPD, also indicated the infants with BPD exhibited a decrease in the microecological diversity of the respiratory tract. In contrast, Lohmann et al. reported that infants with BPD had significantly more *Proteobacteria* (mainly γ Proteobacteria), and significantly less *Firmicutes* and *Fusobacteria* (especially Lactobacillus) than those without BPD (*P* < 0.002). Differences in study results may be related to the clinical characteristics of the enrolled infants, methodology, or the ecological environment of the institute. In Lal et al., bacterial imbalances were observed in the five extremely low birth weight infants with BPD. These infants exhibited decreased abundance of *Firmicutes*, represented by *Lactobacillus*, as well as increased abundance of *Proteobacteria*. A decrease in *Lactobacillus* content may be associated with the occurrence and development of BPD. Animal experiments have reported improved alveolar development in mice transplanted with *Lactobacillus*, indicating that *Lactobacillus* may regulate alveolar development and prevent the occurrence of BPD (37). At the phylum level of the respiratory microbiome, there was no significant difference between infants who developed BPD and those who did not (*P* = 0.18). However, at the genus level, the amount of Lactobacillus in infants with BPD was lower than that in infants without BPD.

### Evolution of Respiratory Tract Microecology and BPD

Wagner et al. reported that preterm infants that eventually developed severe BPD had a lower colonization rate of Staphylococcus in the respiratory tract after birth. In the days after birth, microecological evolution of the respiratory tract became more obvious, and the MH value—which represents the evolution of the microecological community such that lower values indicate faster evolution—was 0.48 in infants with severe BPD, which was higher than that observed in infants with mild BPD. In addition, the weekly detectable microflora load in respiratory tract specimens of infants with moderate and severe BPD was higher than that in infants with mild BPD. These results indicate that the colonization pattern and evolution of the respiratory microbiome in preterm infants may be a marker for predicting the severity of BPD (13).

Imamura et al. also observed changes in *Corynebacterium* species (Cs) content in the lower respiratory tract of infants with severe BPD. Among the 169 extremely premature infants included in the study, 102 did not develop severe BPD, while 67 eventually developed severe BPD. The results indicated that longer ventilation time was associated with higher rate of Cs detection in the lower respiratory tract in infants with severe BPD (12). Cs widely distributes in the environment, belongs to gram-positive bacteria, which is part of the normal flora of human skin and respiratory mucosa. However, in the last few years, Cs has been recognized as a conditional pathogen that causes a variety of infections in immunodeficient hosts, of which *Corynebacterium amycolatum* and *Corynebacterium jeikeium* are important pathogens. Moreover, *Corynebacterium striatum* has also been reported to exhibit potential pathogenicity in patients with chronic diseases (38). However, further large-sample studies are required to determine whether Cs can lead to severe BPD, especially in very premature infants.

Due to the influence of objective conditions such as different research methods and the clinical characteristics of participants, different researchers have offered different conclusions. As seen in Table 1, Imamura et al.'s study was a case-control study, while others were cohort study. And studies of Lohmann and Mourani had a small sample size relatively. In addition, up to now, most studies' objects are neonates underwent endotracheal intubation after birth, which may cause selection bias. However, their studies have all confirmed the influence of microecological changes in the respiratory environment on the risk of BPD in premature infants. At present, the long-term impact of changes in respiratory microecology in infants with BPD are still unclear, and additional studies are required to clarify the correlation between the two. In recent years, studies have mainly included preterm infants who have undergone postnatal endotracheal intubation, while the development of respiratory microorganisms in infants who have not undergone endotracheal intubation, such as those receiving oxygen through continuous positive airway pressure or high-flow nasal catheters, remains unclear. Newer and more effective detection techniques, such as metagenomics, can improve the identification rate of respiratory specimens and may aid in drawing similar conclusions regarding changes in the microbial community associated with respiratory diseases.

### Ureaplasma and BPD

Ureaplasma is a common pathogenic bacterium in the respiratory tract of premature infants and includes both *Urealyticum urealyticum* and *Ureaplasma parvum*, which belong to *Firmicutes*. *Ureaplasma* infection during pregnancy is the main cause of *Ureaplasma* colonization in the neonatal respiratory tract. The relationship between *Ureaplasma* infection during pregnancy and BPD in premature infants remains controversial (39). In an early prospective study Payne et al. studied, 55 cases of extremely low and very low birth weight preterm infants. In the 48 preterm infants who survived to the corrected gestational age of 36 weeks, *Ureaplasma* colonization (especially *Ureaplasma parvum*) in the respiratory tract was closely associated with the occurrence of BPD. In their study, *Ureaplasma* positive infants tended to require longer mechanical ventilation than their negative counterparts (*P* = 0.01) and had a greater frequency of BPD (odds ration [OR] = 4.80, 95% confidence interval [CI]: 1.15–20.13, *P* = 0.029), with moderate and severe BPD being more predominant (*P* = 0.01). The results also suggested increase in the colonization of *Mycoplasma humani* in the respiratory tract of infants with BPD, although whether this increase is associated with the occurrence and development of BPD remains to be confirmed (8). In a study of nine premature infants who developed BPD, Mourani et al. also reported that *Urealyticum urealyticum* and *Ureaplasma parvum* could be detected in their early postnatal respiratory secretions. Even if *Urealyticum* was not detected in the early samples, its dominance could also be found in later samples. Although, the dominant microorganisms in the samples obtained at different sampling times differed, the main microorganisms were *Ureaplasma* or *Staphylococcus* (9). This is in accordance with the results of Wagner et al. (13).

In addition, studies have shown that the prophylactic application of azithromycin can reduce the incidence and mortality of BPD by improving the clearance rate of UU in infected infants, and inhibit the pulmonary inflammatory response (40). But a recent meta-analysis, which included five randomized clinical trials, showed that the application of azithromycin had low-quality evidence in reducing BPD or death in preterm infants with positive UU, but not in all preterm infants (41). And the neonatal dysbiosis of azithromycin application should also be considered, the consequences of which might have long-lasting effects on gut microbiota and will increase the susceptibility to autoimmune diseases and inflammatory bowel disease (42). Wei et al. reported that the use of azithromycin could cause the reduction in observed richness and Shannon diversity. Microbiota composition was shifted primarily in the Actinobacteria phylum, especially a reduction of abundance in the genus Bifidobacterium (43). Parker et al. (44) also reported that the use of azithromycin could induced a decline in microbiota richness, and a reduction in the relative abundance of Proteobacteria and Verrucomicrobia. Although there is still no standard for the initial timing, optimal dose, course of treatment, and safety of azithromycin application, several azithromycin therapy for chronic lung disease of prematurity (AZTEC) studies are ongoing (45, 46).

Although the results of the above studies suggest that respiratory *Ureaplasma* colonization is closely associated with the development of BPD in premature infants, some scholars believe that there is no correlation between them. A meta-analysis conducted by Zheng et al. showed that the colonization of *Ureaplasma* was not a risk factor for BPD. This study analyzed the results of 81 studies on *Ureaplasma* and BPD, among which 11 studies reported no significant correlation between *Ureaplasma* and BPD (47). The different results may be related to the different sample sizes and methods of analysis. In addition, studies have reported that early application of erythromycin has no obvious effect on *Ureaplasma* infection (48).

## Roles of Respiratory Tract Microecology in the Pathogenesis of BPD

### Roles of Microbial Metabolites in BPD

Collins et al. speculated that imbalances in the respiratory tract flora may lead to a drop in beneficial bacteria in the microecological community or excessive growth of detrimental bacteria, thereby increasing the risk of BPD. They also suggested that these changes can lead to an inflammatory reaction to oxygen exposure, which may also increase the risk of BPD (38). Microbial communities can affect cell function by generating metabolites, such as short chain fatty acids or tryptophan metabolites, etc. The catabolism of tryptophan occurs under the action of indoleamine 2, 3-dioxygenase 1, an Aryl hydrocarbon receptor agonist. The activation of Aryl hydrocarbon receptors can mediate the immunosuppressive response and promote the development of regulatory T cells through the production of interleukin-22 (IL-22). *Lactobacillus* is known to metabolize tryptophan into Aryl hydrocarbon receptor agonists, and its beneficial effects with regard to tryptophan metabolites have been shown to inhibit inflammation and promote lung health (49). Another study by Lal et al. also found that the airway metabolome of infants developing BPD was enriched for metabolites involved in fatty acid activation and androgen/estrogen biosynthesis when compared with that of infants without BPD. In their study, infants with BPD exhibited increases in lipid metabolites (e.g., estrogen, androgens) in respiratory microorganisms when compared to infants without BPD. Such changes may increase airway inflammation and lead to the occurrence of BPD (50).

In addition, Gentle et al. (51) reported that infants with BPD had significantly lower nitrate reductase (NR) activity than those without BPD at 29 weeks of post menstrual age. NR is an enzyme expressed by several facultative anaerobes in the microbiota over the tongue dorsum crypts. NR containing bacteria catalyze the reduction of nitrate to nitrite. Nitrite can synthesize nitric oxide (NO) *via* protonation within the further 1-electron reduction or acidic gastric environment through a number of hypoxia sensitive enzymes and proteins in the blood and tissues (52). Gentle et al. suggested a latent mechanistic link between the development of BPD and deficiency in NR-activity and related NO-signaling at the corrected gestational age of 29 weeks. Notably, targeting NO-signaling has been explored as a therapeutic strategy for the treatment of BPD. Although oral NR-activity may become a more sensitive indicator for predicting BPD, further studies are required to evaluate whether NO formation and NR-dependent nitrite is indeed altered at 29 weeks of post menstrual age and how this may influence the development of lungs.

### Roles of Microbiome-Induced Inflammation and Immunity in BPD

In addition to non-pathogenic “symbiotic bacteria,” colonization of viruses and pathogenic bacteria in the respiratory tract can cause respiratory tract infection in premature infants, resulting in inflammatory reactions and lung damage, which may eventually lead to BPD. Both respiratory syncytial virus and rhinovirus colonization lead to severe lower respiratory tract infections that damage lung tissue and lead to chronic lung disease (53). A study conducted by (54) found that the rate of respiratory syncytial virus infection was significantly higher in infants with BPD than in infants without BPD (55). Mourani et al. (9) also believed that early colonization of or exposure to neonatal respiratory pathogens can affect the development of neonatal lung immune function and increase the risk of early or late infection, which may be associated with the subsequent development of BPD. Together with other BPD risk factors like mechanical ventilation and hyperoxia, infection triggers the release of a series of pro-inflammatory substances including IL-6, IL-1β, IL-8, tumor necrosis factor alpha (TNF-α), NLR family pyrin domain containing 3 (NLRP3), and collagen I. These inflammatory mediators restrict the activity of vascular endothelial growth factor and surfactant proteins in the immature lungs of premature infants. They also conduce to vascular alterations and influence the development of alveolar and other characteristic pathologies in BPD (1). Toll-like receptor binding-induced reactive oxygen species activate the NLRP3/caspase-1 pathway, that promotes the production of IL-1b to amplify the inflammation process (56). Microbiota dysbiosis can also rise the lipopolysaccharide level, that in turn induces Toll-like receptor and nuclear factor kappa-light-chain-enhancer of activated B cells (NF-κB) to produce IL-6, IL-1, IL-18, IL-4, interferon gamma (IFN-γ), tumor growth factor beta (TGF-β), and TNF-α, which are involved in the pulmonary immune response (57). Furthermore, lung microbiota can alter the function of alveolar macrophages, dendritic cells (DCs), invariant natural killer T cells, Treg cells, and lung-resident Tgd cells to induce neutrophil migration and intervene in inflammation (58).

Studies by O'Dwyer et al. (59) have shown that lung microbiota dysbiosis can trigger the release of pulmonary fibrosis associated pro-inflammatory cytokines, such as IL-6, IL-1β, macrophage inflammatory protein 1α (MIP-1α), IL-12p70, and chemokine ligand 8 (CXCL8). Among them, alveolar IL-6 is positively correlated with the relative abundance of the *Firmicutes* phylum, while IL-12p70 is negatively correlated with the relative abundance of the *Proteobacteria* phylum. Similarly, in another study, O'Dwyer et al. reported that TNF-β, IL-13, IFN-α2, and TNF-α levels decrease with a reduction in the relative abundance of Firmicutes, while IL-4 and IL-13 levels increase with an increase in the relative abundance of *Bacteroidetes* (60). The pathogenesis of BPD is complex, and studies regarding the mechanism of microecological changes in the respiratory tract leading to BPD are limited. Currently, lung inflammation is considered to be the core mechanism.

### Roles of Microbial Dysbiosis of the Gut-Lung Axis in BPD

Intestinal microecology is closely related to respiratory microecology. The lungs and intestine are both derived from the foregut during embryonic development, and both are characterized by a strong correlation between mucosal immunity and tissue development. Studies have shown that some immune cells in the intestinal mucosa, such as CD4+ and CD8+ cells, can simultaneously appear in both the intestine and lung. Mucosal immunity may constitute an immune network between the lungs and intestines (61). Animal experiments have indicated that the intestinal microflora can regulate the immune response in distant mucous membranes, and that the mortality rate of respiratory virus infection increases when the gastrointestinal microecology is disturbed (62). Children with asthma exhibit alterations in respiratory microflora, and the composition of their intestinal microflora also differs from that observed in healthy children through stool sampling (63). Chen et al. reported that infants with BPD are more likely to develop gut dysbiosis in early life. In their study, the BPD group had a significantly lower diversity of gut microbiota than the preterm group on day 28 (*P* = 0.034) (6).

**Figure 1:**
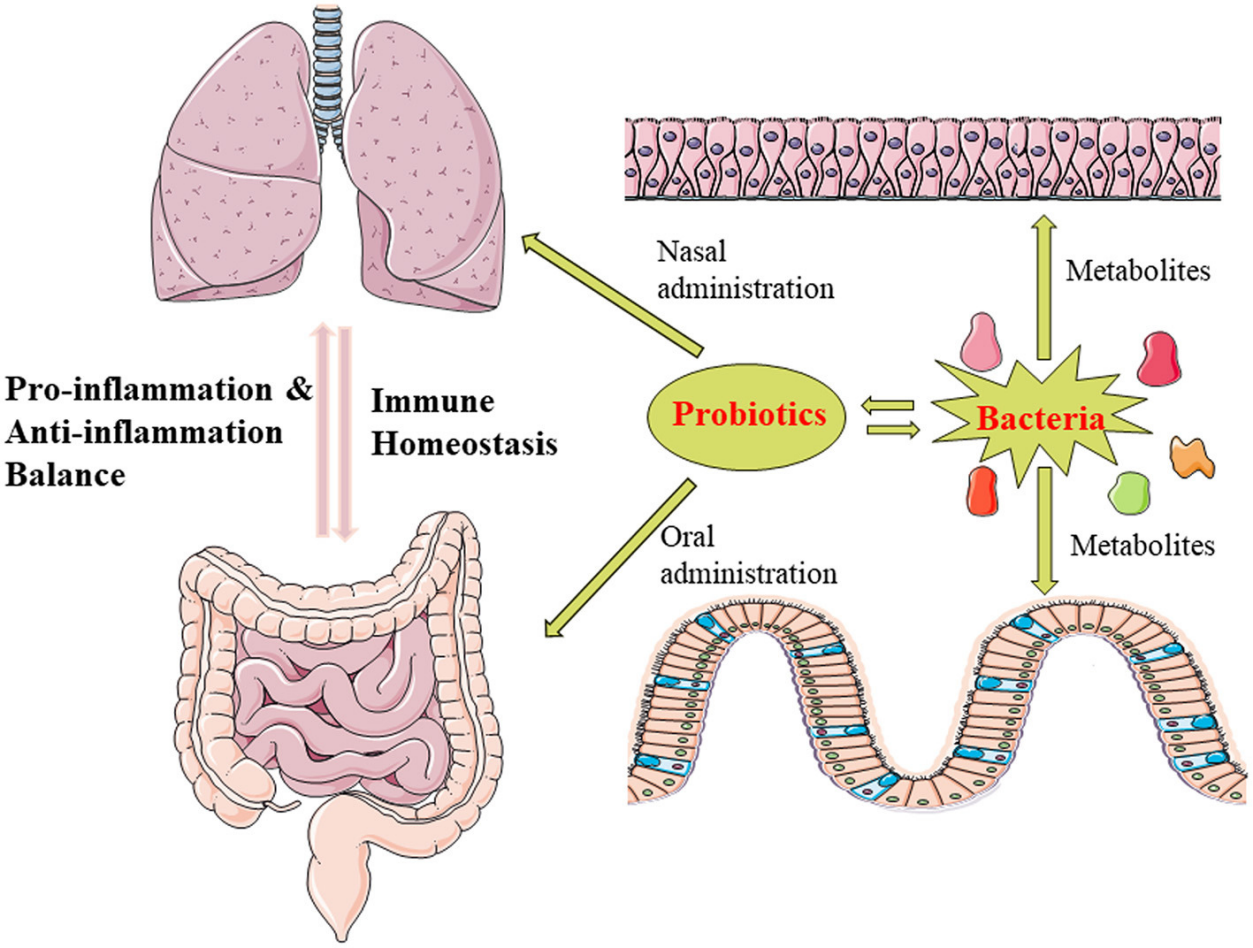
The lung and gut exhibit close cross-talk through microbiota, and the concept of the gut-lung axis has been proposed. Microbiota can influence amino acid and lipid metabolism, and the resultant metabolites can regulate the release of pro-inflammatory and anti-inflammatory cytokines, helping to maintain immune homeostasis between the gut and lung. Further, administration of some probiotics can reduce both pulmonary and intestinal inflammation, which may promote lung development and aid in the prevention and treatment of BPD. (The illustrations are provided by Servier Medical Art (https://smart.servier.com/) licensed under a Creative Commons Attribution 3.0 Unported License).

Some scholars have also proposed the concept of the gut-lung axis (Figure 1), that involves complicated cross-talk between gut/lung disease and lung/gut microbiota dysbiosis. The concept has been widely tested and appears vital for immune response and homeostasis in the airways (7, 64). Ryan et al. proposed that the composition of the gut microbiota may affect the risk of developing BPD and shape systemic immune gene expression. In their research, the gut microbiota of infants (born at <29 weeks gestation) with and without BPD differed significantly. Levels of immune gene expression are also significantly altered in infants with BPD, suggesting a correlation between immune gene expression in the blood and the composition of the microbiota in early life (65). Animal studies have shown that the gut-lung axis has a previously unrecognized effect on the development of neonatal lung injury. Such findings may aid in ameliorating the most severe and persistent pulmonary complications in preterm infants. Willis et al. reported that the interruption of perinatal intestinal commensal colonization promoted a more severe BPD phenotype associated with increased mortality and pulmonary fibrosis. Mechanistically, metagenomic metastasis has been correlated with downregulation of IL-22 expression in bronchoalveolar lavage, but not with hyperoxia-induced inflammasome activation (66). Furthermore, gut microbiota can also modulate BPD susceptibility by changing trimethylamine N-oxide levels (67). Accumulating evidence from recent studies indicates that the microecological environment of the respiratory and intestinal tracts can influence each other, and that both can influence the development of BPD. Microbiota dysbiosis in these organs can lead to immune disorders and exacerbate the disease outcome *via* triggering the inflammatory process. Hence, the findings suggest that preserving the microecological balance in the lungs and intestines can significantly improve BPD.

### Roles of the Microflora-Exosome-miRNA Regulatory Network in BPD

**Figure 2:**
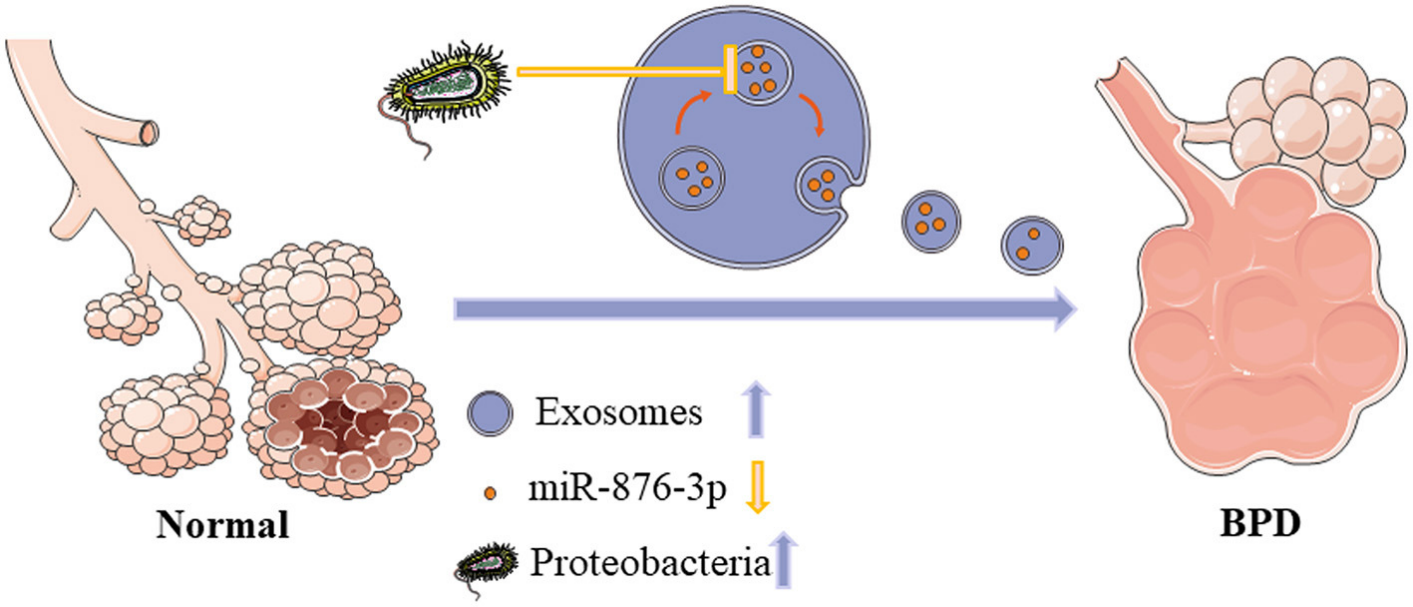
Roles of the microflora-exosome-miRNA regulatory network in BPD. Exosomes participate in intercellular communication and pathophysiological processes. In severe BPD, tracheal aspirates contain more exosomes, although there is decreased expression of miR-876-3p decrease, which may represent a biomarker for the prediction of severe BPD. Moreover, *Proteobacteria* are more abundant in infants with severe BPD and can inhibit the expression of miR-876-3p. Therefore, the microflora-exosome-miRNA network should be considered as a tool for BPD prediction and a target for BPD treatment. BPD: bronchopulmonary dysplasia.

Recent studies have confirmed the roles of exosomes and miRNAs contained within exosomes in cell differentiation and tissue and organ development. Such studies have also indicated that miRNAs play an important role in lung development and lung injury (68). Another study conducted by Lal et al. found that increased exosomes content in the airway secretions of preterm infants (born at <28 weeks of gestation) with severe BPD, although the expression of miR-876-3p in exosomes was decreased. These findings suggest that low expression of miR-876-3p can be used to predict the occurrence and development of BPD in preterm infants. The authors also noted that *Proteobacteria* can regulate the expression of miR-876-3p (Figure 2). In the BPD model, *Proteobacteria* can increase the release of exosomes but reduce the expression of miR-876-3p, thus inhibiting alveolar development and leading to the occurrence and development of BPD. This study demonstrated the presence of a respiratory tract microbiota-exosome-miRNA regulatory network in the pathogenesis of BPD, which may aid in predicting the development of BPD in premature infants (69).

### Potential Applications of the Respiratory Tract Microbiome in BPD Treatment

Similar to the role of intestinal probiotics in the prevention and treatment of intestinal diseases, researchers have increasingly focused on the role of respiratory probiotics in the treatment of lung diseases. Probiotics are live microorganisms that have a beneficial influence on the health of the host when given in sufficient doses, with minimal adverse effects and high safety. (70) reported that prophylactic administration of probiotics could reduce ventilator-associated pneumonia in mechanically ventilated children (70).

The possible mechanism of probiotics in either prevent and/or treat BPD may associated with its anti-infection and anti-inflammatory ability. At present, most studies have focused on *Lactobacillus*, which exhibits a strong anti-inflammatory effect. *Lactobacillus* can also increase the number of regulatory T cells in the lungs and promote alveolar development. An earlier animal study also showed that nasal administration of *Lactobacillus* reduces airway inflammation, highlighting its potential in the treatment of BPD and other lung diseases (71). Another animal study indicated that oral administration of *Lactobacillus plantarum* could promote regulatory T cell immunoregulatory action by decreasing the number of macrophages, neutrophils, and pro-inflammatory cytokines. In their study, oral administration of *Lactobacillus plantarum* significantly weakened pulmonary inflammation in mice infected with *Klebsiella pneumoniae* (72). In addition, as we all know, oxidative stress plays a central function in BPD pathogenesis, probiotics also have the antioxidant ability. *Lactobacillus* and *Bifidobacterium* are known to possess antioxidant properties (73). Yun et al. (37) also reported that *Lactobacillus* translated into the lungs of germ-free mice alters alveolar structure and regulates alveolar growth. Although microbiota such as *Lactobacillus* may exert protective effects in the lungs, no studies have demonstrated this protective role in animal models of chronic lung disease in neonates.

In addition, due to the regulatory mechanism of the human lung-gut axis, some intestinal probiotics that can improve gastrointestinal symptoms have been shown to reduce the occurrence of BPD in premature infants (7). Studies have reported decreased incidence of BPD when *Bifidobacterium* was used to prevent necrotizing enterocolitis in premature infants, although further studies are required to improve the efficacy of *Bifidobacterium* alone in the treatment of BPD (74). The use of probiotics opens up a new era of clinical treatment, but there are still some unanswered questions regarding their use, such as the manner of use, the age, and dose at which they should be administered, and the long-term prognosis of patients, following their use. Nonetheless, there may be a latent role of respiratory tract probiotics in the future.

## Conclusion

The etiology and pathogenesis of BPD are complex, and effective strategies for prevention and treatment remain lacking. Studies suggest that changes in respiratory tract microecology can affect the occurrence and development of BPD in premature infants, mainly including the decreased diversity, faster evolution and UU infection. The roles of respiratory tract microecology in the pathogenesis of BPD mainly including microbial metabolites, microbiome-induced inflammation and immunity, microbial dysbiosis of the gut-lung axis, and microflora-exosome-miRNA regulatory network. Clarifying the specific alterations in bacterial species within the microenvironment of the respiratory tract is expected to lead to new targets for therapeutic intervention in patients with BPD. Although probiotics are beneficial to humans, their use in neonates with BPD remains unproven. There are several limitations in this review, including the small sample size of the studies cited, limited studies on term or preterm infants, and different study methods. It's hard to draw a unified conclusion. Due to the lack of studies on respiratory tract microecology and BPD during the neonatal period, further studies are required to identify the mechanism by which the two interact.

## Author Contributions

JF conceived the review and revised the manuscript. TS performed the search, collect the data, and took charge of writing the original manuscript. HY drew the figures. All authors contributed to the article and approved the submitted version.

## Funding

This research granted from the funding: Key R&D Guidance Plan Projects in Liaoning Province (2020JH1/10300001).

## Conflict of Interest

The authors declare that the research was conducted in the absence of any commercial or financial relationships that could be construed as a potential conflict of interest.

## Publisher's Note

All claims expressed in this article are solely those of the authors and do not necessarily represent those of their affiliated organizations, or those of the publisher, the editors and the reviewers. Any product that may be evaluated in this article, or claim that may be made by its manufacturer, is not guaranteed or endorsed by the publisher.
